# Proof-of-Concept Study: Hyperspectral Imaging for Quantification of DKK-3 Expression in Oropharyngeal Carcinoma

**DOI:** 10.3390/bioengineering12090971

**Published:** 2025-09-12

**Authors:** Theresa Mittermair, Andrea Brunner, Bettina Zelger, Rohit Arora, Christian Wolfgang Huck, Johannes Dominikus Pallua

**Affiliations:** 1 Institute of Pathology, Neuropathology, and Molecular Pathology, Medical University of Innsbruck, Muellerstrasse 44, 6020 Innsbruck, Austria; theresa.mittermair@aon.at (T.M.); andrea.brunner-veber@innpath.at (A.B.); bettina.zelger@i-med.ac.at (B.Z.); 2 Innpath GmbH, Tirolkliniken, Anichstrasse 35, 6020 Innsbruck, Austria; 3 Department of Orthopaedics and Traumatology, Medical University of Innsbruck, Anichstraße 35, 6020 Innsbruck, Austria; rohit.arora@i-med.ac.at; 4 Institute of Analytical Chemistry and Radiochemistry, University of Innsbruck, Innrain 80–82, 6020 Innsbruck, Austria; christian.w.huck@uibk.ac.at

**Keywords:** immunohistochemistry, hyperspectral imaging, digital image analysis, oropharyngeal squamous cell carcinoma, DKK-3

## Abstract

Introduction: Oral squamous cell carcinoma (OSCC) is one of the most common tumours worldwide. This study investigated the suitability of visible and near-infrared hyperspectral imaging compared to visual assessment and conventional digital image analysis for quantifying immunohistochemical staining on the example of Dickkopf-3 (DKK-3) in OSCC. Materials and methods: A retrospective analysis of TMAs containing DKK-3 stained OSCC of 50 patients was retrieved from the archives at the Institute of Pathology, Medical University of Innsbruck. TMAs were first evaluated visually, followed by digital image analysis using QuPath (version 0.3.2, open-source software). For hyperspectral imaging, six exemplary cases were selected (three cases with strong expression and three cases with weak expression) and evaluated. The collected hyperspectral images were visualised using TIVITA (Tissue Imaging System). The resulting true-colour images and the classified HSI images were then assessed using the QuPath software. The Allred score and the H-score were used for all analyses. Results: 97 tissue cores were used for visual and digital image analysis. No significant difference was found between the evaluations of visual and digital image analysis using the H-score (pWilcoxon = 0.278), and both H-scores correlated significantly with each other (p^Spearman^ < 0.001). Similar results were also found using the Allred score. The kappa value was 0.67, which represents a “substantial” correlation. Finally, the H-scores and Allred scores were compared for visual, digital, and HSI imaging. No significant differences were found between the three groups concerning the H-score (pWilcoxon > 0.1). Using Cohen’s Kappa, a “fair” to “moderate” correlation was observed between the three evaluations. Conclusion: Visible and near-infrared hyperspectral imaging (VIS-NIR-HSI) is a promising complementary tool for digital pathology workflows. This proof-of-concept study suggests that HSI offers the potential for more objective quantification of DKK-3 expression in oropharyngeal squamous cell carcinoma, particularly in cases with weak staining. However, given the small sample size and exploratory design, the findings should be regarded as hypothesis-generating. Future studies with larger, clinically annotated cohorts and standardised workflows are needed before any consideration of routine clinical application.

## 1. Introduction

Oral squamous cell carcinoma (OSCC) represents one of the most common malignancies worldwide, accounting for more than 90% of all oral cancers, with significant global morbidity and mortality [1]. Despite advances in surgical and adjuvant therapies, the 5-year survival rate remains low, mainly when diagnosis occurs at an advanced stage [2]. Early detection and precise molecular characterisation are crucial for enhancing clinical outcomes and informing targeted therapies.

One promising biomarker in OSCC is Dickkopf-3 (DKK-3), a member of the Dickkopf family that acts as a modulator of the Wnt signalling pathway. DKK-3 plays a pivotal role in regulating cellular proliferation, differentiation, and apoptosis [3,4]. Interestingly, while DKK-3 has been recognised as a tumour suppressor in various cancers, such as prostate and gastric carcinomas [5], recent studies suggest an oncogenic role of DKK-3 in head and neck squamous cell carcinoma, with higher expression levels correlating with poorer prognosis [6]. This dual role underlines the importance of accurately quantifying DKK-3 expression in tissue specimens, which can provide crucial insights into tumour biology and patient prognosis.

Although widely applied, traditional immunohistochemistry (IHC) is subject to inter-observer variability and lacks the capacity for objective quantification of marker expression, particularly when distinguishing subtle differences in expression. Recent advances in digital pathology have enabled semi-automated analyses; however, these techniques are still dependent on complex segmentation algorithms and may struggle in highly heterogeneous tumour environments [7,8]. Hyperspectral imaging (HSI) emerges as an innovative modality, offering spatially and spectrally resolved datasets that capture biochemical tissue properties without needing labels or stains [9]. The unique ability of HSI to generate a molecular fingerprint of tissues via visible and near-infrared (VIS-NIR) spectral data enables non-destructive, objective quantification of molecular markers, such as DKK-3 [10]. HSI has already demonstrated significant potential in oncology, allowing for differentiation between malignant and benign tissues, the identification of tumour margins, and even prediction of therapeutic responses [11,12]. Yet, its application to quantitative immunohistochemical analysis, particularly for potentially clinically relevant biomarkers such as DKK-3 in OSCC, has not been fully explored. In this study, we aimed to assess the applicability of VIS-NIR-HSI for quantifying DKK-3 expression in OSCC. We compared HSI-based analysis to conventional visual assessment and digital image analysis using open-source software (QuPath). By correlating HSI data with traditional IHC scoring systems, including Allred and H-scores, we evaluated the potential of HSI to serve as a novel diagnostic and prognostic tool in routine pathology. The aim of this proof-of-concept study was not to replace traditional IHC evaluation but to explore whether HSI could provide complementary, objective information—particularly in cases with weak staining, where conventional methods may struggle. Given the moderate correlation achieved, this work should be viewed as proof of concept. Our results provide essential insights into the role of HSI in augmenting traditional pathology workflows and its prospects for future clinical integration. This study was designed as a technical feasibility analysis focusing on analytic concordance between visual, digital, and HSI-based quantification methods. It did not attempt to correlate DKK-3 expression with clinical outcomes such as tumour stage, HPV status, treatment response, or survival. For consistency and comparability, we employed the Allred and H-score systems, which are widely applied in IHC research beyond their original use in breast cancer receptors. While not explicitly validated for DKK-3 in OSCC, its established structure enables exploratory benchmarking across visual, digital, and HSI-based assessments.

## 2. Materials and Methods

### 2.1. Sample Collection and Reassessment of Diagnosis

This study used tissue microarrays (TMAs) containing formalin-fixed, paraffin-embedded (FFPE) samples of OSCC. These samples were previously collected from 50 patients diagnosed between 1990 and 1997 at the Institute of Pathology, Medical University of Innsbruck. Dickkopf-3 staining was then performed during a pilot study on TMAs, including 97 punches from 50 patients. For our research, archived slides of these TMAs were used retrospectively to conduct a comparative analysis. TMAs were re-evaluated for diagnosis, and non-representative samples were excluded from further evaluation. All tissue samples were fully anonymised, and the study was approved by the local ethics committee (EK: 1087/2022), following the Declaration of Helsinki.

### 2.2. Visual and Digital Image Analysis

DKK-3 immunohistochemical staining was visually evaluated independently by two experienced pathologists (AB, BZ) under a light microscope (Olympus BX50) at a magnification of ×200. Both pathologists were blinded to the results of digital image analysis and HSI evaluation to minimise observer bias. In cases of discrepancy, a consensus was reached in joint review sessions. Each TMA tissue core (punch) was evaluated based on tumour cell-specific DKK-3 staining. Non-tumour areas (e.g., stromal cells, inflammatory infiltrates) were excluded from the scoring to ensure tumour specificity.

Two established semi-quantitative scoring systems were applied:

- H-score: This score reflects the percentage of tumour cells stained at different intensities. It is calculated using the formula:

$$H-score=\left(\%weah\times 1\right)+\left(\%moderate\times 2\right)+(\%strong\times 3)$$

Thus, the H-score ranges from 0 (no staining) to 300 (100% intense staining).

- Allred score: This score combines two sub-scores—the proportion score (PS) for the percentage of positive tumour cells and the intensity score (IS) for staining intensity. The total score ranges from 0 to 8.

Detailed scoring criteria are provided in Supplementary Table S1. Although both scoring systems were originally developed for evaluating breast carcinoma hormone receptors, they are widely applied as semi-quantitative metrics across various tumour entities. In this study, their use ensured consistent comparison between visual, digital, and HSI-based quantification. However, they have not yet been explicitly validated for DKK-3 expression in OSCC.

### 2.3. Digital Pathology (Digital Image Analysis–DIA)

**Figure 1:**
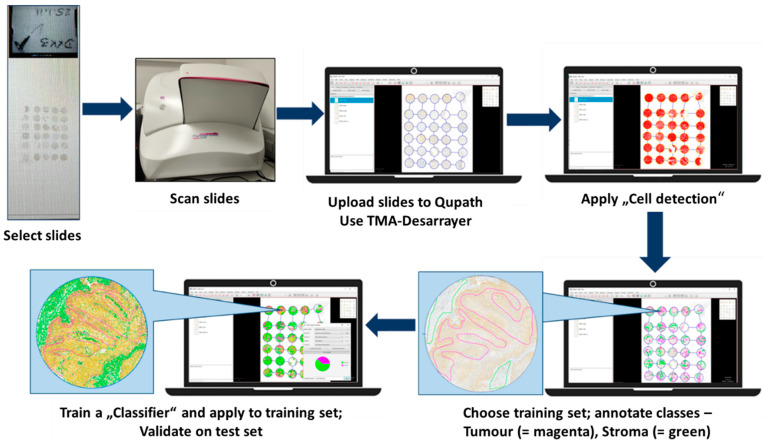
Workflow of digital image analysis (QuPath). From immunohistochemical staining of DKK-3 on TMAs to high-resolution whole-slide scanning and AI-based analysis with QuPath. The pipeline includes tumour annotation, classifier training, cell segmentation, and quantitative analysis (H-score and Allred score).

To complement and objectify the visual evaluation, digital image analysis (DIA) was employed using QuPath (version 0.3.2), an open-source platform for quantitative histopathological image analysis. All immunohistochemically stained TMAs were scanned at high resolution using a 3DHistech Pannoramic 250 Flash whole-slide scanner, generating standardised digital images suitable for detailed computational analysis. Following digitisation, regions of interest (ROIs) corresponding exclusively to tumour areas were manually annotated in QuPath to ensure accurate segmentation of tumour tissue while excluding stromal, necrotic, or inflammatory components (Figure 1). For automated tissue classification and DKK-3 quantification, a Random Tree classifier, integrated within QuPath, was trained on representative annotated TMAs. This classifier differentiated between the tumour and non-tumour compartments, categorising tumour cells as positive or negative, and further categorised staining intensity into weak, moderate, or strong [7,13]. Cell segmentation and classification were performed using QuPath’s machine learning-based cell detection algorithms, which identified nuclei based on size, shape, and immunohistochemical staining intensity (using the DAB chromogen). The resulting data included absolute cell counts, the percentage of positive cells, and categorised staining intensities, allowing for the subsequent calculation of both the H-score and the Allred score, as defined in the visual scoring system [14]. All digital outputs were subjected to expert review by the pathologists who had performed the visual assessment to ensure consistency and address discrepancies. This review process was essential for validating the classifier’s performance and maintaining clinical relevance in the digital quantification process [15]. By utilising this AI-assisted, machine learning-based approach, the study achieved standardised and high-throughput quantification of DKK-3 expression in OSCC. The implementation of DIA provided an objective comparison to visual assessments and established a crucial reference framework for evaluating the novel HSI-based analysis performed in parallel. However, reproducibility was not assessed in this study—no repeat measurements, inter-operator testing, or independent cohort validation was performed. Future studies must address these points to confirm robustness. Moreover, this method underscores the growing significance of digital pathology and artificial intelligence (AI) in routine diagnostic workflows and biomarker research, thereby contributing to enhanced reproducibility and reduced observer bias [16,17]. However, it should be noted that the Random Tree classifier was trained on manually annotated ROIs only, and no independent cross-validation, external test set, or inter-observer reproducibility analysis was performed. This reflects the exploratory, proof-of-concept nature of the present study.

### 2.4. Hyperspectral Imaging (HSI)

To explore the potential of HSI as a novel tool for the quantitative evaluation of immunohistochemical (IHC) staining, we applied visible and near-infrared (VIS-NIR) HSI to analyse DKK-3 expression in selected OSCC cases. HSI data were acquired using the TIVITA^®^ Tissue System (Diaspective Vision GmbH, Pepelow, Germany), a camera system that allows high-resolution spectral imaging in the 500–1000 nm range, coupled to an Olympus PROVIS AX 70 microscope (Olympus Corporation, Ina Plant, Ina, Japan) via a standard C-mount interface. Before image acquisition, all infrared filters were removed from the optical path to ensure unrestricted spectral data collection across the desired range. The TIVITA system was operated under standardised illumination conditions, utilising a halogen light source to transmit homogeneous light through the stained tissue sections. Dark and bright reference measurements were performed before each imaging session to correct for background noise and light intensity variations, ensuring accurate reflectance spectra [9,10]. The more prominent tissue microarray dataset selected six representative cases (three with visually determined high DKK-3 expression and three with low expression) for HSI analysis, providing a focused set for proof-of-concept evaluation. Due to the technical limitation of the current HSI system, which does not allow whole-slide imaging, analysis was restricted to representative ROIs. This approach may not fully capture intratumoural heterogeneity and should therefore be considered a limitation of the present study. Tissue sections were imaged at 20× magnification to capture detailed morphological and spectral features. Each HSI dataset, or “spectral cube,” consisted of two spatial dimensions (x, y) and one spectral dimension (λ), providing a comprehensive dataset of the tissue’s spatial and spectral properties [11,12]. To prevent external light contamination during image acquisition, slides were analysed in a fully shielded environment, and all measurements were performed within minutes to avoid temporal variations in illumination. The entire acquisition process, including calibration, took approximately 10 s per image, demonstrating the feasibility of rapid HSI scanning in routine pathology settings [18].

### 2.5. HSI Data Processing and Analysis

The acquired HSI datasets were processed using the TIVITA Suite (version 1.6.0.1) Tissue software, which provides both true-colour images and false-colour visualisations based on spectral features. For each region of interest (ROI), defined by prior histopathological annotation, average spectral signatures were extracted, capturing the reflectance profiles of tumour cells stained for DKK-3. To distinguish tissue compartments and identify areas of positive DKK-3 expression, two main analytical approaches were applied:

1. Principal Component Analysis (PCA): PCA was used to reduce the dimensionality of the HSI data while preserving most of the variance. This method allowed for unsupervised spectral classification, separating tumour tissue, background, and DKK-3-positive regions based on their spectral signatures [19,20]. The first three principal components were used to generate false-colour composite images, which visualised tissue heterogeneity.
2. Correlation Functions (CFs): CF analysis was applied to compare the spectral signatures of unknown regions with those of reference spectra from DKK-3-positive cells. This method enabled pixel-wise tissue classification based on spectral similarity, allowing the spatial distribution of DKK-3 expression to be mapped without additional labelling [21].

**Figure 2:**
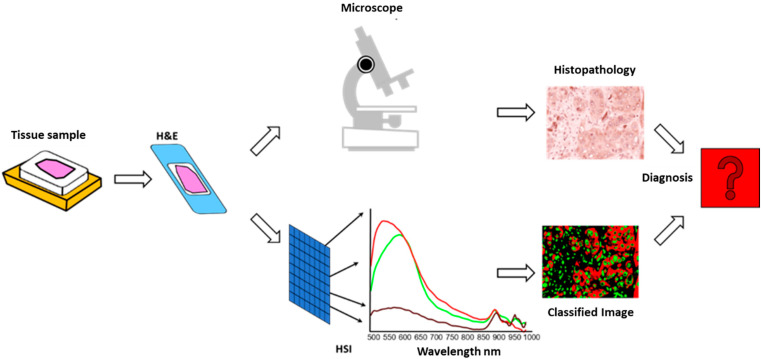
Conceptual workflow of hyperspectral imaging (HSI)-based analysis for quantification of DKK-3 expression in oropharyngeal squamous cell carcinoma. Formalin-fixed paraffin-embedded (FFPE) tissue samples are sectioned, stained for DKK-3, and analysed using a hyperspectral imaging system coupled to a microscope. The acquired hyperspectral datasets are processed using TIVITA Suite Tissue software to generate true-colour and false-colour images and spectral signatures. Regions of interest (ROIs) are defined based on histopathological annotation. Two analytical approaches are applied for spectral analysis: (1) Principal Component Analysis (PCA) for unsupervised classification and visualisation of tissue compartments based on spectral variance, and (2) Correlation Functions (CFs) for pixel-wise classification of DKK-3-positive regions by comparing unknown spectra with reference signatures. The resulting classified images and spectral data are imported into QuPath for further analysis using standardised immunohistochemistry (IHC) scoring systems (Allred and H-score). This integrative approach allows for objective quantification of DKK-3 expression, supporting direct comparison with conventional visual and digital image analysis methods.

The resulting false-colour images and classified spectral maps were exported for further analysis and comparison with traditional IHC evaluations. To ensure methodological consistency, the HSI-derived data were imported into QuPath and analysed using the same scoring systems applied in visual and digital evaluations (Allred and H-score). This multimodal integration directly compared HSI, visual, and digital quantification. A conceptual workflow of the HSI-based diagnostic process is illustrated in Figure 2.

### 2.6. Statistical Analysis

All statistical analyses were conducted using IBM SPSS Statistics (version 26.0 for Windows) and Microsoft Excel (2016). The Shapiro–Wilk test was applied to assess the normality of score distributions for visual, digital, and HSI-based assessments. Due to significant deviations from normality (*p* < 0.001), non-parametric tests were employed throughout the analysis. The Wilcoxon signed-rank test was used to compare paired differences between the evaluation methods (visual, digital, HSI). This analysis detected systematic differences in DKK-3 expression scores across methods. The correlation between different scoring approaches was assessed using Spearman’s rank correlation coefficient (ρ), which provides a measure of the association between the quantitative outcomes of visual, digital, and HSI analyses.

Additionally, Cohen’s Kappa statistic was calculated to evaluate the level of agreement between categorical scores derived from visual, digital, and HSI assessments. Kappa values were interpreted as follows: values between 0.00 and 0.20 indicate slight agreement, 0.21 to 0.40 fair agreement, 0.41 to 0.60 moderate agreement, 0.61 to 0.80 substantial agreement, and values above 0.81 near-perfect agreement [22]. A *p*-value < 0.05 was considered statistically significant for all tests. The 95% confidence intervals (CIs) were provided where appropriate to support key findings. This robust statistical framework allowed for a comprehensive evaluation of methodological concordance and assessment of HSI as a potential diagnostic tool in OSCC.

## 3. Results

Three TMAs with 30 oral squamous cell carcinoma tissue samples and one TMA with 16 samples were analysed. Nine tissue samples were excluded from the study because they did not contain sufficient tumour tissue. Thus, 97 tissue samples were analysed visually and using digital image analysis and HSI imaging for this study. The results of the visual evaluation and digital image analysis are provided in Supplementary Table S2.

**Figure 3:**
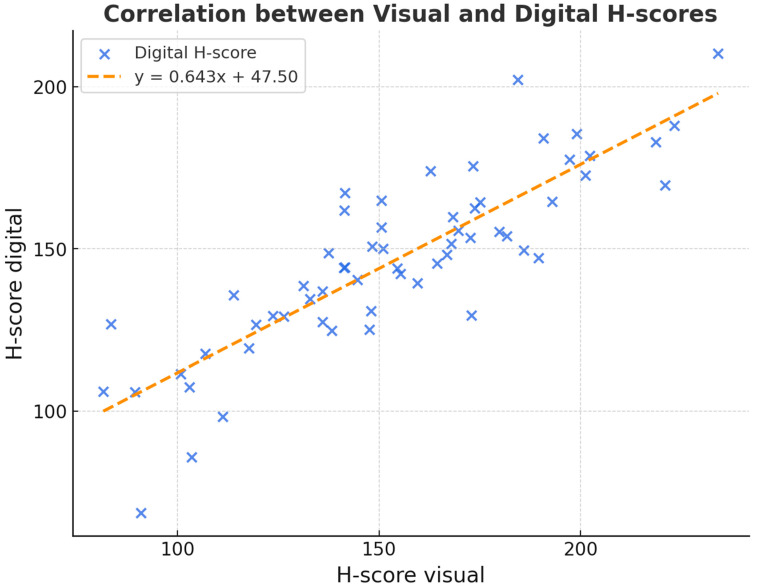
Scatter plot of the relationship between the digitally and visually determined H-score of DKK-3 expression in squamous cell carcinoma of the oropharynx.

No statistically significant differences were observed between visual and digital scoring (Wilcoxon *p* = 0.278). This suggests broad comparability, although formal equivalence cannot be claimed, as no predefined equivalence margins or power analysis was performed. Both H-scores nevertheless correlated significantly with each other (pSpearman < 0.001). The following scatter plot (Figure 3) shows the relationship between the digital and visual H-scores to determine DKK-3 expression.

Similar results were also obtained using the Allred score for DKK-3 expression (see Table 1).

The kappa value was 0.67, meaning a “substantial” correlation exists between the two analyses. This indicates moderate concordance between visual and digital image analysis, but reproducibility was not directly assessed. Furthermore, we could not determine any association of DKK-3 expression with the degree of differentiation, although the central mass of the tumours corresponded to a grade 2 carcinoma, as expected.

### 3.1. Hyperspectral Image Analysis

**Figure 4:**
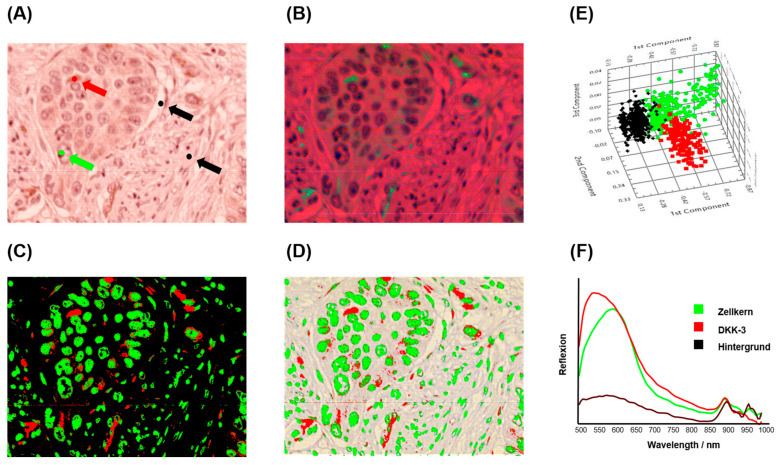
HSI analysis of DKK-3 immunohistochemically stained tissue section. (**A**) Brightfield RGB image of a DKK-3 stained tissue section at 20× magnification showing the defined regions of interest (ROIs): nuclei (green), DKK-3 positive staining (red), and background (black, including stroma and glass slide). (**B**) The Principal Component Analysis (PCA) is a false-colour image representing the first three components (PC1—red, PC2—green, PC3—blue). (**C**) PCA-based tissue segmentation colour-coded for nuclei (green), DKK-3 (red), and background (black). (**D**) Overlay of PCA segmentation and original RGB image to illustrate the spatial distribution of nuclei and DKK-3 signals. (**E**) PCA segmentation focuses on the spectral range of 520–725 nm for enhanced differentiation of tissue components. (**F**) Mean spectral profiles of the defined ROIs (nuclei—green, DKK-3—red, background—black), ranging from 500 to 1000 nm. The HSI analysis enables the distinct separation of DKK-3 expression from the nuclei and background, as validated through both PCA modelling and spectral characterisation.

HSI analysis was performed on six representative cases of DKK-3 immunohistochemically stained tissue sections to evaluate marker expression and tissue morphology at a spectral level. Figure 4 illustrates a representative example of the HSI evaluation workflow. The brightfield RGB image (Figure 4A) highlights the defined regions of interest (ROIs) used for spectral classification, including DKK-3-positive areas (red), nuclei (green), and background regions (black). Principal Component Analysis (PCA) was applied to the spectral data set, generating a false-colour image (Figure 4B) that visually separates tissue components based on their spectral signatures. The PCA-based segmentation (Figure 4C) provides clear and visually reassuring discrimination between DKK-3 expression, nuclear regions, and background. To ensure spatial alignment and visualisation, the segmented image was overlaid on the original RGB image (Figure 4D). Further analysis within the 520–725 nm spectral range (Figure 4E) enhanced the separation of DKK-3-positive staining from nuclear structures, leveraging the specific absorption and scattering features of the stain. The mean spectral profiles for each ROI (Figure 4F) revealed distinct spectral fingerprints: DKK-3-positive areas exhibited higher absorbance in the 520–650 nm range, consistent with the chromogenic stain. At the same time, the nuclei and background showed lower and spectrally distinct responses. Quantitative evaluation across all six cases yielded a mean HSI–H-score of 174.75 (95% CI: 132.5–216.6), a median of 171.7, and a minimum and maximum of 146.34 and 233.65, respectively. The HSI–Allred score averaged 7 (95% CI: 6.4–7.7), and the mean percentage of DKK-3-positive tumour area was 88.31% (95% CI: 82.7–93.9). These results demonstrate that HSI, combined with PCA-based segmentation, provides a robust, quantitative method for assessing biomarker expression in complex tissue environments.

**Figure 5:**
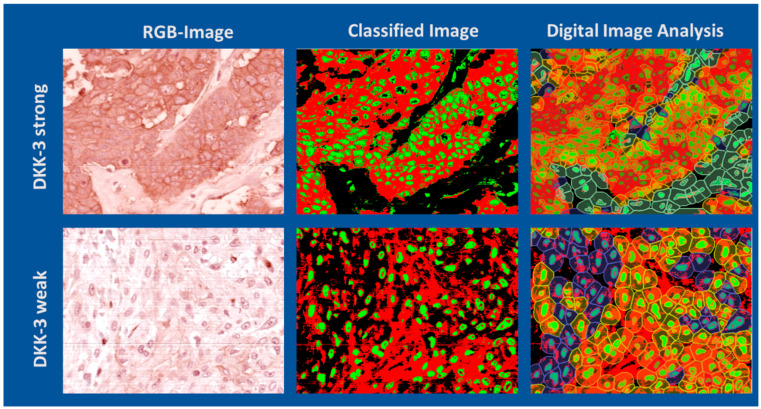
HSI and digital image analysis of oral squamous cell carcinoma with strong and weak DKK-3 expression. Top row: ROI with strong DKK-3 expression represented as RGB image, classified image following HSI data analysis, and image analysis results showing nuclear and cytoplasmic segmentation. Bottom row: ROI with weak DKK-3 expression represented as RGB image, classified image from HSI analysis, and corresponding image analysis with cellular segmentation. The HSI-based classification and image analysis clearly distinguish between high and low DKK-3 expression patterns.

To compare different evaluation methods for DKK-3 expression, regions of interest (ROIs) with strong and weak staining intensities were analysed using HSI, classical digital image analysis, and conventional visual assessment. Figure 5 illustrates examples of ROI with strong (top row) and weak (bottom row) DKK-3 expression, each shown as an RGB image, HSI-classified image, and subsequent image analysis of cellular and extracellular regions. The HSI-based classification enabled the objective discrimination of DKK-3-positive and -negative regions, including the differentiation of nuclei and cytoplasm, independent of staining variability. The subsequent segmentation analysis provided detailed morphometric and intensity-based quantification of DKK-3 expression at a cellular level.

**Figure 6:**
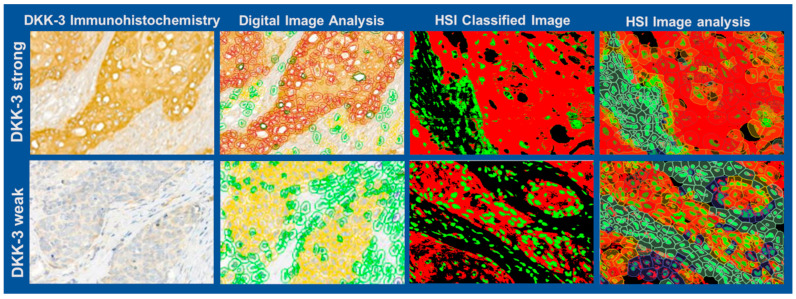
Comparative analysis of DKK-3 expression using visual scoring, classical digital image analysis, and HSI with subsequent image analysis shown for a tumour with high and low DKK-3 expression. Left: DKK-3 immunohistochemistry; second column: classical digital image analysis; third column: HSI-based classified image; right: HSI-based image analysis with cellular segmentation. The figure highlights consistent segmentation and intensity-based analysis for DKK-3 expression using all three methods.

Figure 6 compares visual scoring, classical digital image analysis, and HSI-based analysis for tumours with high (top) and low (bottom row) DKK-3 expression. HSI-based and digital image analyses provide more refined discrimination of expression patterns, particularly in cases with weak staining, compared to visual estimation. The comparison of H-scores and Allred scores obtained by visual, digital, and HSI-based analysis across six representative cases is summarised in Table 2. The HSI analysis yielded a mean H-score of 174.57 (range: 123.78–233.65) and a mean Allred score of 7 (range: 6–8), which were broadly in the range of visual assessment (mean H-score 206.67, mean Allred score 7) and digital image analysis (mean H-score 123.38, mean Allred score 6). HSI provided more consistent quantification, especially in cases with low DKK-3 expression. These results demonstrate that HSI, combined with advanced image segmentation, offers a robust and objective alternative to conventional methods, reducing observer variability and enabling precise quantification of biomarker expression in heterogeneous tumour tissues.

### 3.2. Correlation and Concordance Analysis of Visual, Digital, and HSI-Based DKK-3 Assessment

**Figure 7:**
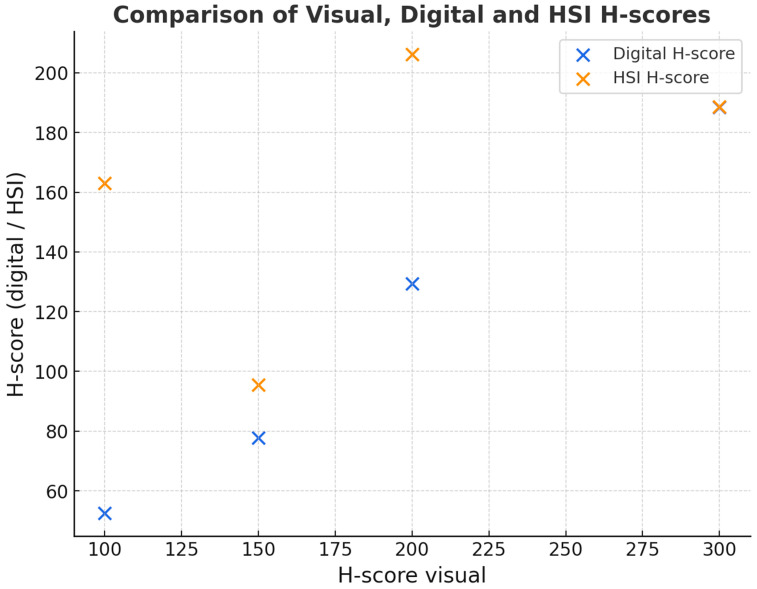
Scatter plot showing the relationship between visual scoring and classical digital image analysis (blue) and between visual scoring and HSI (orange). Linear regression lines are included for both comparisons, indicating a stronger alignment of HSI with visual assessments.

To evaluate the relationship and agreement between different assessment methods for DKK-3 expression, H-scores obtained from visual estimation, classical digital image analysis, and HSI were analysed. Figure 7 illustrates the correlation between visual assessment and digital image analysis (blue dots and line) and between visual assessment and HSI (orange dots and line). The regression for HSI (ρ = 0.67) demonstrates only a moderate correlation with visual scores. While this indicates feasibility, it also highlights that HSI does not replicate visual scoring exactly and should be regarded as complementary rather than a replacement. This suggests that HSI measurements align more closely with visual assessments than classical digital image analysis.

**Figure 8:**
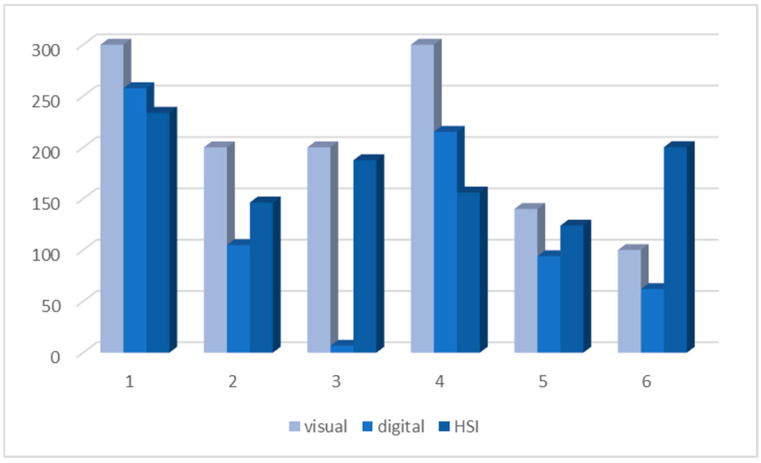
Bar chart comparing DKK-3 expression (H-score) across six oral squamous cell carcinoma cases using visual assessment, classical digital image analysis, and HSI.

Figure 8 presents a bar chart comparing DKK-3 H-scores for the six cases evaluated visually, digitally, and via HSI. While visual scoring tended to yield higher H-scores overall, HSI frequently identified expression levels closer to the visual estimation than digital analysis, particularly in cases with lower expression (cases 5 and 6). Interestingly, although the methods exhibited different tendencies in individual cases, no statistically significant differences were identified between the groups using the Wilcoxon signed-rank test (*p* > 0.1 for visual/digital, visual/HSI, and digital/HSI comparisons).

Table 3 summarises the Cohen’s Kappa statistics based on categorised Allred and H-scores to assess the agreement between the methods. Scores were dichotomised for this purpose: Allred scores into “low” (score 0–6) and “high” (score 7–8), and H-scores based on whether they fell below or above the overall mean H-score. The analysis revealed fair to moderate agreement among all three evaluation methods. Interestingly, HSI showed slightly higher agreement with visual scoring (κ = 0.42 for Allred and H-scores) than classical digital image analysis (κ = 0.40). This suggests that while HSI does not perfectly replicate visual assessment, it may offer a more robust and observer-independent alternative than classical digital approaches.

## 4. Discussion

HSI is a powerful and tissue-preserving imaging technique that objectively evaluates IHC markers. This study aimed to assess the suitability of HSI compared to visual assessment and conventional DIA for quantifying DKK-3 expression in OSCC. Digital analysis software (QuPath, an open-source platform) was also employed to evaluate the HSI images, thereby integrating HSI within established digital pathology workflows. Our results demonstrate that HSI can effectively quantify IHC markers such as DKK-3 following appropriate calibration, sample preparation, and data analysis procedures. As previously highlighted, quantification of IHC markers remains essential in cancer diagnostics and pathology to improve diagnostic precision and inform therapeutic decisions [23]. Comparing HSI-derived data with visual and digital assessments revealed only a moderate correlation (ρ ≈ 0.67), which limits convergence between methods. This finding suggests that HSI cannot replace visual or digital scoring but may complement them by providing objective information in cases of weak staining. Such observations are preliminary, based on a limited dataset, and should be regarded as hypothesis-generating. Importantly, HSI tended to yield more consistent evaluations in weakly stained cases, where visual and digital analyses are most limited. However, it is important to stress that these results are preliminary and should be regarded as part of a proof-of-concept evaluation rather than as evidence for clinical applicability. These findings align with studies demonstrating the successful quantification of markers using HSI in other cancer types [24,25].

Furthermore, in our research, the HSI imaging process, performed using the TIVITA Tissue System in conjunction with an Olympus PROVIS AX 70 microscope, was found to be user-friendly, and HSI images were acquired in less than ten seconds. Nevertheless, this work must be interpreted strictly as a proof of concept. The exploratory design, together with the small number of analysed cases, does not allow any conclusion regarding clinical applicability. Most HSI imaging systems, including ours, remain at an early developmental stage, and complete spectral imaging of entire TMAs is technically challenging due to limitations in spatial resolution and scan area. Several limitations must therefore be acknowledged. First, our analysis relied on the Allred and H-scores, which were initially developed for evaluating hormone receptors in breast cancer. Although these scores are frequently applied across tumour types for semi-quantitative IHC assessment, their analytical validity for DKK-3 in OSCC has not yet been demonstrated. Future studies should therefore include biomarker-specific validation of scoring approaches. A further limitation is that HSI evaluation was restricted to small ROIs rather than whole-slide imaging. As a result, tumour heterogeneity may not have been fully represented. Future HSI platforms with whole-slide imaging capabilities will be essential for reducing this bias and enabling comprehensive tissue evaluation. It is also important to note that HSI was applied to only six representative cases. Thus, the study should be regarded as hypothesis-generating, and larger, clinically annotated cohorts will be required for validation.

Moreover, our study was limited to the analytic concordance between visual, digital, and HSI-based assessments. No correlation was found with clinical outcomes, such as tumour stage, HPV status, treatment response, or survival. This limitation reflects both the exploratory nature of the study and the use of archival TMAs, which lack complete clinical follow-up data. Therefore, the present work should be understood as limited to analytic concordance, without providing prognostic or diagnostic value. Future studies using larger, clinically annotated cohorts will be necessary to establish the prognostic and predictive value of HSI-based biomarker quantification.

Additionally, the Random Tree classifier used for digital image analysis was trained only on manually annotated ROIs and was not subjected to cross-validation, external test sets, or inter-observer reproducibility assessment. This lack of independent validation limits the generalisability of our findings. Future work should incorporate robust validation strategies to ensure reproducibility and clinical applicability. First, HSI evaluation was limited to six representative cases, constituting a relatively small cohort. Although other studies with somewhat larger sample sizes have also reported favourable results [24,25,26], future investigations with expanded cohorts are warranted to validate and generalise these findings.

Additionally, HSI analysis was limited to selected regions of interest (ROIs) rather than entire TMAs owing to insufficient resolution for whole-slide imaging. Previous studies have reported similar limitations, such as those of Willenbacher et al. (2020), who emphasised the need for next-generation HSI systems capable of scanning and analysing entire histological slides or TMAs [24]. Such advancements could significantly reduce selection bias and better address tissue heterogeneity, a known challenge in pathological diagnostics. Moreover, future research should explore the development of standardised workflows and spectral data processing pipelines. Although HSI holds immense potential for biochemical characterisation of tissues through light–tissue interaction analysis, significant hurdles remain regarding its clinical implementation. Notably, current HSI instrumentation is costly, and standardised data acquisition and analysis protocols are lacking, which limits clinical applicability.

Furthermore, HSI’s vast amounts of data require substantial storage capacity and sophisticated processing tools. Future solutions may involve the implementation of data compression algorithms and targeted spectral acquisition to reduce data loads and associated costs [27]. Another important aspect highlighted by this study is the influence of tissue staining quality on image analysis. Archived TMAs were used in this study, and some specimens exhibited uneven staining, primarily due to pre-analytical factors that cannot always be controlled. It is well known that differences in hematoxylin and eosin (H&E) staining exist between laboratories, affecting both visual and digital analyses [28,29,30]. Therefore, developing standardised staining and colour normalisation protocols for digital and HSI pathology is crucial. Recent efforts in digital staining approaches, including AI-assisted spectral transformation and enhancement, have shown promise in reducing colour variability and highlighting molecular features not captured by traditional staining [31,32]. Such digital staining methods could facilitate future objective tissue evaluations. Artificial intelligence (AI) also plays a crucial role in DIA and HSI workflows, which are essential for extracting meaningful information from complex tissue samples. Our study used QuPath for DIA, employing a Random Tree algorithm to classify and distinguish between tumour and non-tumour cells. This machine learning-based approach enabled reliable quantification of DKK-3 expression in TMAs. Similarly, the HSI data were processed using Principal Component Analysis (PCA), which reduces the complexity of spectral datasets and facilitates the clear visualisation of tumour-specific markers. The integration of AI is indispensable for handling the large datasets generated by HSI and extracting diagnostically relevant features. Moving forward, interdisciplinary collaboration between medical experts and computational scientists will be essential to advancing AI-driven HSI methods further and addressing potential scepticism regarding AI use in clinical practice [33,34,35]. AI-driven image analysis, both in HSI and in DIA, offers substantial benefits for improving diagnostic reproducibility, efficiency, and accuracy—significant considerations in light of the increasing diagnostic workloads and the need for personalised medicine approaches [36]. The combination of HSI and AI may provide valuable additional insights into tissue biochemistry and tumour heterogeneity that are not accessible through conventional methods. At this stage, however, these findings should be regarded as exploratory, and further validation in larger cohorts is essential before clinical integration.

## 5. Conclusions

This study demonstrates the feasibility of using HSI as a complementary tool for the quantitative evaluation of immunohistochemical markers in oropharyngeal squamous cell carcinoma. Our findings suggest that HSI yields broadly consistent results with visual and digital image analyses, with the potential for improved objectivity and sensitivity, particularly in weakly stained cases. Validation in larger, outcome-linked cohorts will be required to determine reproducibility and clinical utility. The ability of HSI to generate spatially resolved spectral fingerprints offers a unique advantage for accurate biomarker assessment in complex tissue architectures. Despite current limitations, including small case numbers and technical constraints in whole-slide imaging, HSI holds promise as a complementary modality in diagnostic pathology. Future developments should enhance spatial resolution, simplify data complexity, and establish standardised workflows to facilitate broader clinical applications. Integrating AI and machine learning will be crucial to fully harness the potential of HSI in cancer diagnostics. At this stage, however, our findings should be regarded as exploratory proof of concept, and further validation in larger, outcome-linked cohorts is essential before any clinical conclusions can be drawn.

## Figures and Tables

**Table 1:** Comparison of visual and digital Allred scores.

		Digital		
		Allred 0–6	Allred 7 and 8	Cohen’s Kappa
Visual	Allred 0–6	63	0	0.677
	Allred 7 and 8	13	21	

**Table 2:** Comparative analysis of DKK-3 expression using visual assessment, digital image analysis, and HSI. The table presents H-scores and Allred scores obtained from three evaluation methods across six cases. Visual assessment provides subjective scoring, while digital image analysis enables semi-automated quantification, and HSI-based segmentation facilitates a more detailed and objective classification of DKK-3 expression.

Case (*n*)	H-Score (Visual)	Allred Score (Visual)	H-Score (Digital)	Allred Score (Digital)	H-Score (HSI)	Allred Score (HSI)
1	300	8	257.62	8	233.65	7
2	200	7	104.81	6	146.34	6
3	200	7	6.77	3	187.492	7
4	300	8	215.13	8	155.988	7
5	140	6	94.01	6	123.78	6
6	100	6	61.97	5	200.2	7
Mean (min.–max.)	206.67 (100–300)	7 (6–8)	123.38 (61.97–257.62)	6 (3–8)	174.57 (123.78–233.65)	7 (6–8)

**Table 3:** The agreement between visual, digital, and HSI assessments of DKK-3 expression was analysed using Cohen’s Kappa statistics. Allred scores were dichotomised: 1 = scores 0–6, 2 = scores 7–8. H-scores were categorised relative to the overall mean (1 = below the mean, 2 = above the mean). The analysis reveals a fair to moderate level of agreement between the methods, with HSI showing slightly better concordance with visual assessment than digital analysis.

Case (*n*)	Allred Visual	Allred Digital	Allred HSI	Kappa (Allred)	H-Score Visual	H-Score Digital	H-Score HSI	Kappa (H-Score)
1	2	2	2	0.40 vis./dig. 0.40 dig./HSI 0.42 vis./HSI	2	2	2	0.40 vis./dig. 0.40 dig./HSI 0.42 vis./HSI
2	2	1	1		2	1	1	
3	2	1	2		2	1	2	
4	2	2	2		2	2	2	
5	1	1	1		1	1	1	
6	1	1	2		1	1	2	

## Data Availability

The data presented in this study are available upon request from the corresponding author.

## Supplementary Materials

The following supporting information can be downloaded at: https://www.mdpi.com/article/10.3390/bioengineering12090971/s1, Supplementary Table S1. Scoring systems used for the evaluation of DKK-3 expression. Supplementary Table S2. DKK-3 expression in oral and pharyngeal squamous cell carcinoma as determined by visual assessment, digital image analysis (DIA), and hyperspectral imaging (HSI).

## Abbreviations

AI	Artificial Intelligence
CF	Correlation Function
CI	Confidence Interval
DAB	3,3′-Diaminobenzidine (Chromogen)
DIA	Digital Image Analysis
DKK-3	Dickkopf-Related Protein 3
FFPE	Formalin-Fixed Paraffin-Embedded
H&E	Hematoxylin and Eosin
HSI	Hyperspectral Imaging
IHC	Immunohistochemistry
IS	Intensity Score (Part of Allred Score)
nm	Nanometre
OSCC	Oral Squamous Cell Carcinoma
PCA	Principal Component Analysis
PS	Proportion Score (Part of Allred Score)
QuPath	Quantitative Pathology (Open-Source Digital Pathology Software)
RGB	Red, Green, Blue (Colour Image Model)
ROI	Region of Interest
SPSS	Statistical Package for the Social Sciences (IBM SPSS Statistics Software)
TMA	Tissue Microarray
VIS-NIR	Visible and Near-Infrared
VIS-NIR-HSI	Visible and Near-Infrared Hyperspectral Imaging
WSI	Whole-Slide Imaging
