# Supplementary material for: Proof-of-Concept Study: Hyperspectral Imaging for Quantification of DKK-3 Expression in Oropharyngeal Carcinoma
Source: Bioengineering (Basel). 2025 Sep 12;12(9):971. doi: 10.3390/bioengineering12090971 (PMC12467325; doi:10.3390/bioengineering12090971)
Supplement: Supplementary file 1 [file bioengineering-12-00971-s001.zip › bioengineering-3870558-supplementary.pdf]

Table S1. Scoring systems used for the evaluation of DKK-3 expression.

	Parameter	Criteria	Score
**Allred -Score**	**Proportion of positive cells (PS)**	None	0
		< 1%	1
		1% – 10%	2
		11% – 33%	3
		34% – 66%	4
		> 66%	5
	**Intensity of staining (IS)**	None	0
		Weak	1
		Moderate	2
		Strong	3
	**Total Score**	Sum of PS and IS	0 – 8
**H-score**	%weak	Multiplied by 1 (%W)	0 – 100
	% moderate	Multiplied by 2 (%M)	0 - 200
	% strong	Multiplied by 3 (%S)	0 - 300
	Total Score	Sum of %W, %M and %S	0 - 300

Table S2. DKK-3 expression in oral and pharyngeal squamous cell carcinoma as determined by digital and visual image analysis. The percentage of positive tumours as determined by both methods is also shown.

Case	Visual Allred	DIA Allred	HSI Allred	Visual H-score	DIA H-score	HSI H-score
1 (High)	7	8	8	240	260	275
2 (High)	6	6	7	210	205	230
3 (High)	5	5	6	180	185	200
4 (Low)	3	4	5	85	90	120
5 (Low)	2	3	4	40	50	85
6 (Low)	0	1	3	0	20	65
